# MicroRNA regulation of unfolded protein response transcription factor XBP1 in the progression of cardiac hypertrophy and heart failure in vivo

**DOI:** 10.1186/s12967-015-0725-4

**Published:** 2015-11-16

**Authors:** Quanlu Duan, Chen Chen, Lei Yang, Ni Li, Wei Gong, Sheng Li, Dao Wen Wang

**Affiliations:** Department of Internal Medicine and the Institute of Hypertension, Tongji Hospital, Tongji Medical College, Huazhong University of Science and Technology, 1095 Jiefang Avenue, 430030 Wuhan, People’s Republic of China

**Keywords:** miRNA, XBP1, Heart failure, VEGF

## Abstract

**Background:**

XBP1 is a key transcription factor of the unfolded protein response in mammalian cells, which is involved in several cardiovascular pathological progression including cardiac hypertrophy and myocardial infarction, but its expression trend, function and upstream regulate mechanism in the development of heart failure are unclear. In the present study, therefore, the potential role of miRNAs in the regulation of XBP1 expression in heart failure was examined.

**Methods and results:**

First, western blots showed that cardiac expression of ER stress marker XBP1 were induced in the early adaptive phase, but decreased in the maladaptive phase in hypertrophic and failing heart, while there was no obvious change of upstream ATF6 and IRE1 activity in this progression. Interestingly, we further found that XBP1 and its downstream target VEGF were attenuated by miR-30* and miR-214 in cardiomyocyte. Moreover, we found that miR-30* was significantly reduced in the early phase of cardiac hypertrophic animal model and in human failing hearts, while both miR-214 and miR-30* were increased in the maladaptive diseased heart, thereby contribute to impairment of cardiac XBP1 and VEGF expression.

**Conclusions:**

These results provide the first clear link between miRNAs and direct regulation of XBP1 in heart failure and reveal that miR-214 and miR-30* synergistically regulates cardiac VEGF expression and angiogenesis by targeting XBP1 in the progression from adaptive hypertrophy to heart failure.

**Electronic supplementary material:**

The online version of this article (doi:10.1186/s12967-015-0725-4) contains supplementary material, which is available to authorized users.

## Background

XBP1 is a key regulator of unfolded protein response (UPR) or endoplasmic reticulum (ER) stress response in the mammalian cell [1]. Under stress condition, ER-resident signal transducers IRE1a (inositol-requiring kinase1) cleaves XBP1 mRNA induced by ATF6 to generate a mature mRNA encoding a highly active transcription factor spliced XBP1 (XBP1s) [2]. As a bZIP transcription factor, XBP1 s subsequently translocates to the nucleus and promotes transcription of genes involved in protein folding and degradation to restore ER homeostasis, including EDEM, p58^IPK^, ERdj4, and HEDJ [3]. Moreover, XBP1 also activates the transcription of a variety of genes involved in cancer cell survival [4], immune cell differentiation [5], glucose homeostasis [6–8] and lipid metabolism [9–11], and autophagic response [12]. Recent studies have shown that XBP1 expression and splicing was involved in VEGF signaling and contributes to endothelial cell proliferation and angiogenesis in ischemic tissues [13]. Importantly, XBP1 s was induced in neonatal rat ventricular myocyte cultures subjected to hypoxia and in the heart during ischemia/reperfusion (I/R) in mice, and exerts robust cardioprotection against I/R injury [14]. More recently, we further documented that XBP1 s play an important role in the regulation of cardiac angiogenesis in mice [15]. Despite these findings implicating a potential cardioprotective potential for XBP1, little is known concerning its mechanism of actions in the development of heart failure.

Previous reports indicate that prolonged ER stress-initiated cardiac myocyte apoptosis may play an important role in cardiac hypertrophy, heart failure [16], and myocardial infarction [16, 17]. In the failing human hearts and the maladaptive mice heart, CHOP was significantly higher than normal control heart, and ablation of CHOP attenuates endoplasmic reticulum-mediated apoptosis and cardiac dysfunction induced by pressure overload [18]. Moreover, upregulation of ER chaperones such as GRP78 and calreticulin was detected in the heart from 1 week after thoracic aorta constriction (TAC), but became weaker at 4 weeks after TAC [16]. But the expression manner of UPR transducer XBP1 in prolonged failing heart is still unclear, especially in maladaptive phage. These reports led us to hypothesize that XBP1 maybe also was dynamic activated in the progression of cardiac hypertrophy and heart failure. Hence, the upstream regulation of XBP1 activation in heart failure should be further elucidated.

MicroRNAs (miRNAs) are a new class of endogenous, small, 19- to 25-nucleotide noncoding RNAs that act as negative regulators of gene expression by inhibiting mRNA translation or promoting mRNA degradation [19, 20]. Although miRNAs are involved in cardiac events, such as conductance of electrical signals, myocardial contraction, heart growth, and morphogenesis [21–23], the roles of miRNAs in the progression of cardiac hypertrophy to heart failure remain to be established. Thus, the present study aimed to identify miRNAs that are potentially relevant upstream regulators of the UPR in hypertrophic and failing heart. Our results have indicated that reduced miR-30* is required for XBP1 activation in the early stages of hypertrophic hearts in vivo and the upregulation of miR-30* and miR-214 inhibit the expression of XBP1 by targeting XBP1 3′ UTR, resulting in VEGF suppression in the maladaptive heart phage.

## Materials

### Ethics statement

The institutional review board of Tongji hospital approved this study. Written informed consents were obtained from all donors. Experiments were conducted according to the principles expressed in the Declaration of Helsinki and the NIH Belmont Report. All animal studies were approved by the Animal Research Committee of Tongji College and were done according to the guidelines of the National Institutes of Health (NIH).

### Materials and reagents

Antibodies against XBP-1, Grp78, ATF6, VEGF, ANP, GAPDH and β-actin were purchased from Santa Cruz Biotechnology Inc (Santa Cruz, CA); and antibodies against P-IRE1 were purchased from Thermo Scientific Pierce Antibodies (Rockford, IL). Non-specific negative control oligonucleotides, antimiRNA, mimics, for miR-214 and miR-30* (miR-30a*, miR-30b*, miR-30c-2*, miR-30d*, miR-30e*) and specific siRNA against rat XBP-1 were obtained from RiboBio (Guangzhou, China). All other chemicals and reagents were purchased from Sigma-Aldrich China Inc. (Shanghai, China), unless otherwise specified.

### Cell lines

The H9c2 (2-1) cells and 293T cells were obtained from the American Type Culture Collection (ATCC, Manassas, VA, USA) and were grown in Gibco DMEM medium supplemented with 10 % fetal bovine serum (Gibco, Invitrogen, Carlsbad, CA, USA). All cells were grown at 37 °C in an atmosphere of 5 % CO_2_.

### Animal models

Eight-week-old male Sprague–Dawley rats, weighing 160–180 g, were subjected to abdominal aorta coarctation (AAC) or sham operation, as described previously [24]. Pressure overload-induced cardiac hypertrophy and heart failure was created by AAC for 1 day, 3 days, 1, 2, 4, 6, 8, and 10 weeks. Other animals were continuously infused with 5 mg/(kg d) isoproterenol hydrochloride (Sigma-Aldrich China Inc, Shanghai, China) or saline, as previously described [25] for 1, 2, and 4 weeks. Rats were anesthetized with pentobarbital sodium at a dose of 40 mg/kg body weight intraperitoneally. Animals were euthanized via an anaesthetic overdose (200 mg/kg of pentobarbital sodium delivered by intraperitoneal injection).

### Vector construction and luciferase reporter assay

The pMIR-REPORT- XBP-1 3′UTR vector was constructed as described previously [26]. The XBP-1 mutant 3′UTR (Mut-3′UTR) were mutated using an Easy Mutagenesis System kit (TransGen Biotech, Beijing, China). For luciferase reporter assays, 293T cells (1 × 10^5^) were plated in a 24-well plate and then co-transfected with 400 ng of pMIR-reporter vector, and 20 ng of pRL-TK, using Lipofectamine 2000 (Invitrogen, Carlsbad, CA), following the manufacturer’s protocol. Firefly and Renilla luciferase activity was analyzed at room temperature in a chemiluminometer (GloMax, Promega), according to the manufacturer’s instructions, using the Dual-Luciferase Reporter Assay System (Promega). For each experiment, relative luciferase activity was defined as the mean value of the firefly luciferase/Renilla luciferase ratios obtained from three independent experiments.

### Real time PCR assay

Total RNA was extracted from harvested cells using TRIzol (Invitrogen, Carlsbad, CA), according to the manufacturer’s instructions. cDNA was synthesized using EasyScript First Strand cDNA Synthesis SuperMix (TransGen Biotech, Beijing, China) and subjected to SYBR Green real-time analysis (TransGen Biotech, Beijing, China) according to the manufacturer’s instructions (MyIQ; Bio-Rad Laboratories). Abundance of target transcripts was normalized to those of a control small non-coding RNA, U6, in the same samples.

### Cell transfection

H9c2 cells were seeded in 6-well plates at 1.5 × 10^5^ cells/well, 24 h before transfection. Cells were transfected after seeding using Lipofectamine 2000 (Invitrogen, Carlsbad, CA), according to the manufacturer’s recommendations. Cells were collected 48 h after transfection for protein and RNA extraction.

### Statistical analysis

The data are expressed as mean values ±SD. Difference between groups were evaluated for significance using Student *t* test of unpaired data or one-way analysis of variance (ANOVA) and Bonferroni post-test. P < 0.05 was considered significant.

## Results

### Dynamic expression of XBP1s in pressure overload and Isoproterenol-induced hypertrophic and failing heart

**Fig. 1:**
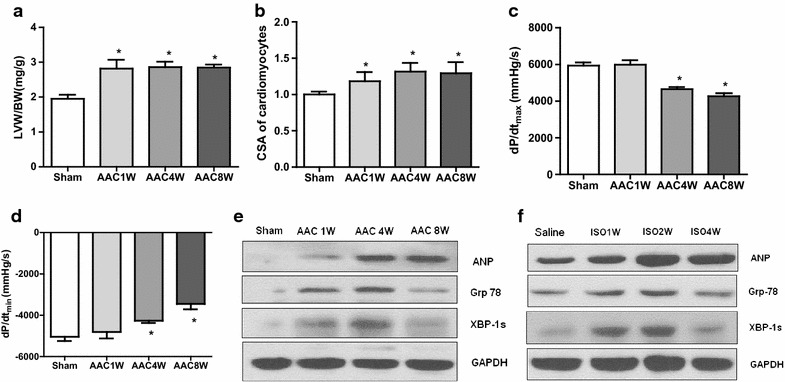
Cardiac XBP1 expression is upregulated in hypertrophic and failing heart. **a** Left ventricular weight/body weight (LVW/BW, grams) after AAC. **b** Cross-sectional area (CSA) of cardiomyocytes after AAC. **c, d** Hemodynamic analysis of rats at 1, 4 and 8 weeks of AAC: (*Upper*) dP/dt_max_ (mmHg/s) and (*lower*) dP/dt_min_ (mmHg/s). **e** Western blots of Grp78, XBP-1 s, and ANP in rat heart after AAC. **f** Western blots of Grp78, XBP-1 s, and ANP in ISO-treated heart samples. n = 6 for **a**, **c, d**; n = 3 for **b**. *P < 0.05 compared with control

A rat pressure overload induced-hypertrophy model was established using AAC. In this model, morphological and hemodynamic analysis demonstrated cardiac hypertrophy gradually developed from 1 to 4 weeks after AAC and decreased thereafter (Fig. 1a–d). Myocardial atrial natriuretic peptide (ANP) protein, a cardiac hypertrophic marker, was also significantly upregulated after AAC treatment (Fig. 1e). Importantly, as depicted in Fig. 1e, protein levels of the GRP78 and XBP-1 were increased as early as 1 week and reached peak at 4 weeks after AAC treatment (Fig. 1e). Both Grp78 and XBP-1 s declined at 8 weeks, suggesting that AAC induced-pressure overload caused aberrant ER stress in the early phase but subsided at 8 weeks after AAC treatment. Isoproterenol (ISO) infusion is another well established model to study hypertrophic and failing hearts. We next assayed the levels of ER stress and XBP-1 s in ISO-induced hypertrophy in rats. Consistently, protein levels of the ER chaperone GRP78 and UPR transcription factor XBP-1 were increased in the early phase and was reduced in the late phase in ISO model, in vivo (Fig. 1f). These findings indicate that the UPR was activated in the early phase of cardiac hypertrophy, from 1 to 4 weeks, but impaired in the late phase, suggesting a potential upstream regulation mechanism for the UPR in the adaptive mechanism of cardiac hypertrophy.

### miR-214 inhibits XBP1 expression and is upregulated in hypertrophic and failing heart

**Fig. 2:**
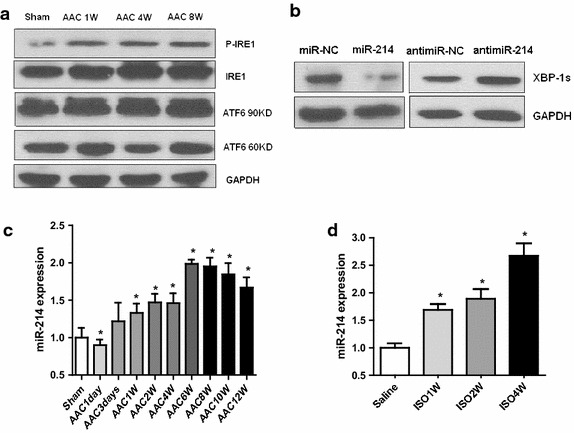
miR-214 inhibits XBP1 expression and is upregulated in hypertrophic and failing heart. **a** Western blots of ATF6 and IRE1 in rat heart after AAC. **b** Western blots of XBP1 s in untreated and miR-214-treated H9C2 (2-1) cells. **c** Real-time PCR analysis of miR-214 in rat heart after AAC treatment. n = 6. **d** Real-time PCR analysis of miR-214 in rat heart after ISO infusion. Values are mean ± SEM. n = 6. *P < 0.05 compared with control

How might XBP1 expression be upregulated during the early phase of cardiac hypertrophy but downregulated in the maladaptive disease heart? As XBP-1 mRNA is induced by ATF6 and spliced by IRE1 in response to ER stress, we first examined the levels of ATF and IRE1 activity. Surprisingly, there was no obvious change of ATF6 and IRE1 activity in hypertrophic and failing rat hearts (Fig. 2a), suggesting there might be alternative mechanism by which XBP-1 was regulated in hypertrophic hearts. MiRNAs are considered a novel regulatory mechanism of gene expression. We hypothesized that miRNAs might be responsible for the upregulation of XBP1 expression in hypertrophic hearts.

Our previous data have shown that XBP1 is a target of miR-214 in hepatocyte and endothelial cell [15]. Furthermore, experiments in H9c2 (2-1) cells showed that ectopic expression of miR-214 caused a significant decrease in the expression of XBP-1 s, while inhibition of endogenous miR-214 by synthetic miR-214 inhibitor resulted in the upregulation of XBP-1s (Fig. 2b). These data suggest that XBP1 also is a target of miR-214 in cardiomyocyte.

Next, to investigate the potential involvement of miR-214 in cardiac XBP1 expression, we performed real time PCR to analyze the changes in myocardial miR-214 expression during development in two established hypertrophy models. As shown in Fig. 2c, d, miR-214 expression was significantly increased in both ISO- and AAC-induced cardiac hypertrophy. Hence, upregulated of miR-214 under prolonged cardiac stress maybe contribute to XBP1 downexpression in maladaptive cardiac diseases. Surprisingly, as a competent regulator of XBP1, why expression of miR-214 was enhanced while expression of XBP-1 still was increased during the early phase of cardiac hypertrophy? We reasoned that there may be another mechanisms regulated XBP1 expression in the pathophysiologic procedures.

### miR-30* targets XBP1 in cardiomyocytes

**Fig. 3:**
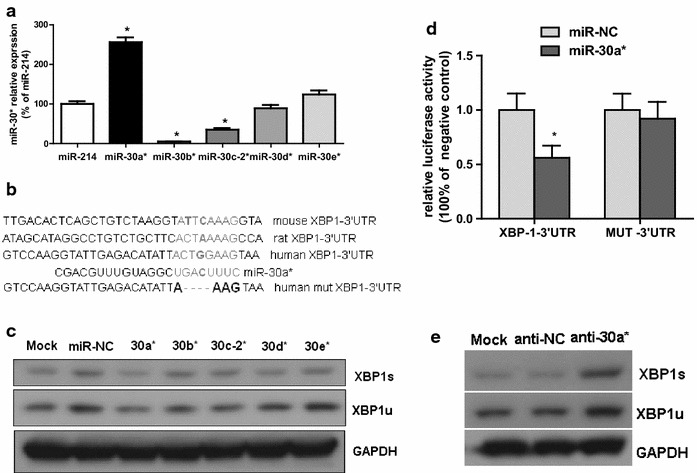
XBP1 is experimentally validated as a direct target of miR-30a*. **a** Expression of the miR-214 and miR-30* family in normal mice heart tissue. n = 6. **b** a schematic diagram of the reporter constructs showing the entire XBP1 3′ UTR sequence and the sequence of the miR-30* binding sites within the human XBP1 3′ UTR and MUT 3′ UTR. **c** Western blot of XBP1 in H9C2 (2-1) cells 48 h after transfection of miR-30* mimics or miRNA negative control (miR-NC) oligonucleotides. **d** Luciferase activity 24 h after transfection of the pMIR-XBP1-3′ UTR reporter or pMIR-XBP1-3′ UTR mut reporter and miRNAs. **e** Western blot of XBP1 in H9C2 (2-1) cells 48 h after transfection of miR30a* inhibitors (antimiR-30*) or miRNA inhibitors negative control (antimiR-NC) oligonucleotides (100 nM). *P < 0.05 compared with miR-NC

To fully understand the mechanisms by which other miRNAs execute their function, two targeted prediction algorithms, MICROCOSM and TargetScan, were utilized to identify the potential miRNAs targeting XBP1. Potential miRNA binding sites in the XBP1 3′UTR were predicted using these tools, and analysis indicated that XBP1 is an evolutionarily conserved target of miR-30*. We predicted six miRNAs for miR-30* (recently designated miR-30-3p) family, including miR-30a*, miR-30b*, miR-30c-1*, miR-30c-2*, miR-30d*, miR-30e* (Fig. 3a), with potential base pair complementarities to conserved sequences in the XBP1 mRNA 3′UTR (Fig. 3b). We first analyzed the effect of the miR-30* family on XBP1 expression and found that among these candidates, overexpression of miR-30a* caused a significant decrease in the protein levels of XBP1 s in H9c2 cells, with greater effect than the others (Fig. 3c). Thus, miR-30a* were selected for further analysis. To measure a direct interaction between miR-30* and its potential binding site within XBP1 mRNA, the pMIR-reporter-XBP1-3′UTR (XBP1-3′UTR) vector or pMIR-reporter -XBP1-3′UTR mut (mut 3′UTR) vector was co-transfected into 293T cells along with miR-30a* mimics or miRNA negative controls (miR-NC) and assayed for expression of a luciferase reporter. XBP1-3′ UTR vector-transfected 293T cells showed a distinct decrease in luciferase activity when co-transfected with miR-30*, while no significant change in luciferase activity was observed following the co-transfection of mut 3′ UTR vector with miR-30* mimics or miR-NC (Fig. 3d). These findings suggest a direct interaction between miR-30* and XBP1 3′ UTR.

Finally, the effect of miR-30* inhibition on the endogenous expression of XBP1 was further examined in H9C2 (2-1) cells. The results showed that inhibition of endogenous miR-30a* by synthetic miR-30a* inhibitor resulted in up-regulation of XBP1 (Fig. 3e). These data suggest that miR-30* can inhibit the expression of XBP1 by directly targeting the 3′-UTR of XBP1 mRNA.

### miR-30* are downregulated in the early phase of cardiac hypertrophy, but restored in maladaptive hypertrophy

**Fig. 4:**
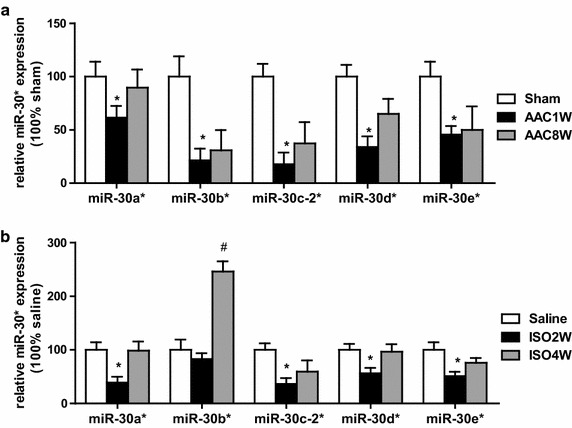
Dynamic expression of miR-30* contributed to XBP1 dysregulation in hypertrophic and failing heart. **a**, **b** Dynamic levels of miR-30* in AAC-treated heart, ISO-induced heart model, respectively. n = 6. *P < 0.05 compared with control

As cardiac expression of XBP-1 was induced in the early adaptive phase, but decreased in the maladaptive phase in hypertrophic and failing heart, it was interesting to monitor the levels of miR-30* in the different phases of AAC-induced cardiac hypertrophy. As shown in Fig. 4a, miR-30a* expression was significantly decreased in the early phase of cardiac hypertrophy at 1 weeks after AAC treatment but was up-regulated at 8 weeks under prolonged stress. XBP1 was declined in response to miR-30* upregulation in maladaptive hypertrophy, further suggesting that miR-30* is able to directly target the XBP1 pathway, either during the period of compensated hypertrophy or during the transition to heart failure. To further establish the relevance of the above observations of miR-30* with ISO infused hypertrophic model, we then analyze the changes in myocardial expression of the miR-30* family and found that the expression trend of miR-30a* in ISO model heart was similar with AAC model (Fig. 4b). Moreover, the time-course change in the ratio of miR-30a*/miR-214 during cardiac hypertrophy and heart failure (Additional file 1: Fig. S1) show that down-regulation of miR-30a* may minimize the role of increased miR-214 in regulation of XBP-1 in the early phase of cardiac hypertrophy, while increased expression of both miR-214 and miR-30* synergistically lead to suppression of XBP1 in the maladaptive heart.

### miR-214 and miR-30* reduce the expression of XBP1’s targets in cardiomyocyte

**Fig. 5:**
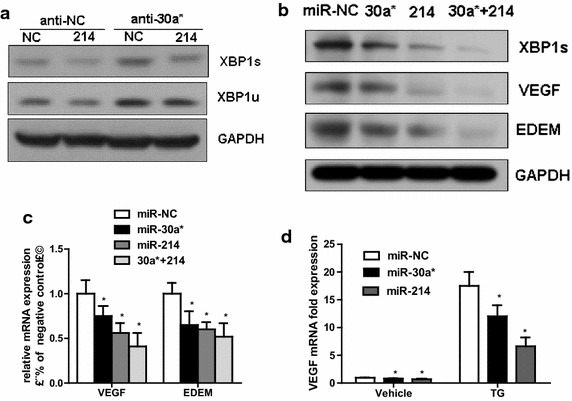
miR-214 and miR-30* reduce the target of XBP1 expression in cardiomyocytes. **a** Western blot analysis of XBP1 in H9C2 (2-1) cells co-transfected with miR-30a* inhibitors (antimiR-30a*) and miR-214 mimics. **b** Western blot analysis of XBP1, VEGF and EDEM in H9C2 (2-1) cells transfected with or without miR-30a* and miR-214 mimics or miRNA negative control oligonucleotides (100 nM). **c** Real-time PCR analysis of VEGF and EDEM mRNA in H9C2 (2-1) cells transfected with or without miR-30a* and miR-214 mimics or miRNA negative control oligonucleotides (100 nM). **d** Real-time PCR analysis of VEGF expression in untreated and TG-treated H9C2 (2-1) cells transfected with or without miR-30a* or miR-214 mimics or miR-NC (100 nM). n = 3. *P < 0.05 compared with control

In the early phase of cardiac hypertrophy, the expression trend of miR-30a* and miR-214 were opposite, while XBP1 was increased, so we treated cardiomyocyte with miR-214 mimics and miR-30a* inhibitors (anti-30a*) to further test if down expression of miR-30a* reverses suppressive effect of miR-214 upregulation in XBP1 expression. The western blot results show that XBP1 reintroduction by transfection of miRNA-30a* inhibitors into miR-214 mimics-transfected cardiomyocyte ablated the effects of miR-214 on XBP1s expression (Fig. 5a).

In the maladaptive phase of cardiac hypertrophy, both miR-30a* and miR-214 were increased, while XBP1 was decreased, so we treated cardiomyocyte with both miR-214 and miR-30a* mimics. Interestingly, a more significant decrease in XBP1 expression were observed in miR-214 and miR-30a* co-transfected cells (Fig. 5b). Furthermore, as a key transcription factor, XBP-1 s can translocate into the nucleus, then binds to its target sequence in the regulatory regions of the downstream genes to induce their transcription such as EDEM and VEGF [27–29]. To further test the relationship of these miRNAs and XBP1, we performed western blot analysis and found that the protein levels of VEGF and EDEM were diminished by miR-214 or miR-30a* overexpression in H9c2 (2-1) cells (Fig. 5b).

Consistently, similar results of real time PCR analysis were also observed in H9c2 (2-1) cells (Fig. 5c). To determine if two miRNAs is involved in VEGF induction through the targeting of XBP1 following UPR activation, we treated H9c2 (2-1) cells transfected with miRNA mimics with or without TG treatment. We found that both miR-214 and miR-30a* mimics decreased VEGF mRNA expression in response to ER stress (Fig. 5d). The VEGF-A suppressing effect of miR-214 and miR-30* over expression was similar to what was measured after down regulation of XBP1 in H9c2 (2-1) cells (Additional file 1: Fig. S2). These results suggest that ectopic expression of miR-214 and miR-30* led to a decrease in XBP1 expression, sequentially inhibited the expression of its targets.

### Reduced miR-30* caused cardiac XBP1 and VEGF upregulation in hypertrophic and failing heart

**Fig. 6:**
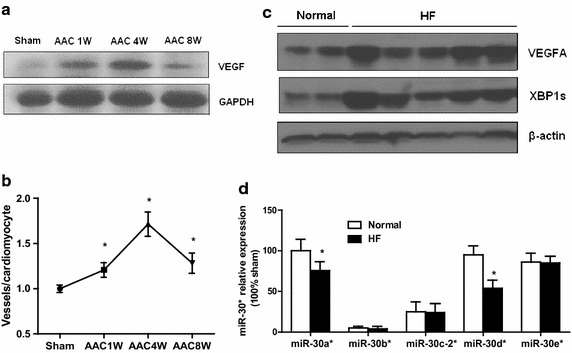
Reduced miR-30* caused cardiac XBP1 and VEGF upregulation in hypertrophic and failing human heart. **a** Western blots of VEGF in rat heart after AAC. **b** Immunohistochemical analysis revealed that the number of CD31-positive cells was increased in hearts after AAC. **c** Western blot analysis of cardiac XBP1s expression in human heart. **d** The expression of miR-30* in human failing heart. n = 6, *P < 0.05 compared with normal control

VEGF is required to maintain myocardial capillary density and that reductions in the vascular bed are associated with the transition from compensatory hypertrophy to failure [30]. Hence, we therefore examined the expression of VEGF, the downstream target of miR-30*/XBP1, in AAC heart. Western blots revealed that cardiac expression of VEGF-A, was correlated with XBP1 s, peaked at 4 weeks after AAC and decreased thereafter in rat hypertrophic and failing heart (Fig. 6a). Consistent with the Western blots, immunohistochemical analysis showed that capillary density was increased in the same manner observed in rat hearts after AAC (Fig. 6b, Additional file 1: Fig. S3).These data show that dynamic expression of miR-30* and miR-214 caused opposite expression of XBP1 and VEGF in hypertrophic and failing heart.

Finally, we measured the expression of miR-30* and XBP-1s in two normal hearts and six patients with heart failure and the results showed that both XBP-1 and its downstream target VEGF were significantly increased in all failing human hearts, with the mean signal intensity increased compared to normal hearts (Fig. 6c). Next we performed real time PCR to analyze the changes in myocardial miR-30* expression in human failing heart tissue. As shown in Fig. 6d, miR-30a* expression was decreased in all failing human hearts when compared to those from normal hearts. So downregulated miR-30a* contribute to that the level of XBP1 was significantly higher in human failing heart tissue.

Together, these results of upregulated miR-214 and reduced miR-30* expression in several forms of heart failure raise the intriguing possibility that disbalance between miR-214 and miR-30* actually cause accumulation of XBP-1 protein in the early phase of cardiac hypertrophy and thereby contribute to impairment of cardiac XBP1 expression in the maladaptive diseased heart.

## Discussion

In our previous study, we found that miR-214 is an important regulator of angiogenesis in endothelial cells regulating the expression of XBP1 in the control of cardiac angiogenesis [15]. Now, we further found that miR-214 regulates XBP1 expression in cardiomyocytes. Indeed, miRNA microarray analysis shows that expression of miR-214 is significant unregulated in TAC- and calcineurin A-induced mouse heart hypertrophy models, as well as in idiopathic end-stage failing human hearts [22, 31]. Real-time PCR analysis confirmed that miR-214 showed a striking increase in expression in the border zone of the infarct, both in the murine and human myocardial infarction hearts [32, 33]. miR-214 is also unregulated following renal ischemia reperfusion injury compared with sham controls [34, 35]. Importantly, miR-214 overexpression appeared to be capable of inducing hypertrophic growth in cardiomyocytes [31]. These reports indicate that distinct miR-214 was regulated during heart failure, suggesting the possibility that it might function as modulators of this process. Previous studies have shown that induces expression of miR-214 that actively downregulate several mitochondrial and cardiac targets including PPARδ, provoking a switch toward a glycolytic metabolic profile that contributes to heart failure [36]. Then we further established the essential roles of miR-214 in hypertrophic and failing heart by down-regulating XBP-1 mediated angiogenesis [15]. Interestingly, in early stage of hypertrophic heart, miR-214 was mildly induced in the early adaptive phase whereas XBP-1 was unregulated instead of being down regulated. Possible explanations included dynamic alterations in mRNA and protein turnover, or structural changes in the 3’UTR accessibility or other microRNAs acting in an opposite direction may have a role during this pathophysiological process [37, 38]. In fact, we further found that miR-30a* expression was significantly decreased in the early phase of cardiac hypertrophy, but return to baseline levels in maladaptive hypertrophy. Furthermore, this discrepancy of miR-214 and XBP-1 levels can be explained by relative low levels of miR-214 and high activity of ATF6, and IRE1 in early stage of hypertrophic hearts. Under ER stress, cleavaged ATF6 induce XBP1 transcription, and phosphorylation of IRE1α induces the splicing of XBP-1 mRNA [2]. In the early stage of hypertrophy, ATF6 cleavage or IRE1α phosphorylation were highly activated in hearts after TAC and ISO infusion. The inhibitory effects of miR-214 on XBP-1 are likely overwhelmed by activated ATF6 or/and IRE1 caused by UPR in hypertrophic hearts. However, along with increasing expression of miR-214 in late stage of hypertrophy, the inhibitory effects of miR-214 become dominant which results in the suppression of XBP-1.

In another hand, we also found that XBP1 is a potential target of the miR-30* family. As we known, the miR-30* family members include miR-30a*, miR-30b*, miR-30c*, miR-30d* and miR-30e* [39]. As with the miR-30 family, miR-30* family members all have the similar ‘‘seed sequence’’ in their 5′ termini, and are abundantly expressed in the heart under physiological conditions [22]. Previous studies shows that expression of miR-30* and miR-30 were significantly down-regulated in mouse heart hypertrophy models and failing human hearts [22, 40]. Real-time PCR analysis confirmed that miR-30* and miR-30 showed a striking decrease in expression in a murine model of right ventricular hypertrophy (RVH) and failure (RVF) pulmonary artery constriction (PAC) [41]. These reports indicate that distinct miR-30* were regulated during heart failure, suggesting the possibility that this might function as modulators of this process. Recent study found that miR-30* family was involved with TGF-beta induced-impaired endothelial cells function [42]. Nonetheless, their function in the heart remains largely unknown. We extend the previously reported loss of mature miR-30* family in hypertrophic hearts [22, 41, 42], to two rodent models of cardiac hypertrophy and heart failure. In particular, we found that restored expression of miR-30* decreased the protein levels of XBP1 s and the mRNA levels of VEGF in H9c2 cells and the luciferase activity of the pMIR-reporter-XBP1-3′UTR (XBP1-3′UTR) vector in 293T cells. A recent study indicated that miR-30c-2* regulates XBP1 expression and the magnitude of XBP1-mediated gene transcription by targeting the 3′UTR of XBP1 [39], results which are in line with our conclusion. In fact, we further found that miR-30a* expression was significantly decreased in the early phase of cardiac hypertrophy, but return to baseline levels in maladaptive hypertrophy. Along with increasing expression of miR-30* in the late stage of hypertrophy, the inhibitory effects of miR-30* on XBP1 become dominant, and results in the suppression of XBP1 and impairment of cardiac angiogenesis. In this study, we have established the essential roles of miR-30* in the transition of the hypertrophic heart to the failing heart by down-regulation of XBP1.

Recently, spliced XBP1 was reported to couples the unfolded protein respon to hexosamine biosynthetic pathway to confers strong cardioprotection against ischemia/reperfusion damage [14]. In our present study, we further found that XBP1 regulate VEGF expression in cardiomyocytes, which is in a line with that XBP1 s can bind to two regions on the VEGFA promoter contributes to VEGFA expression in response to ER stress in human cancer cells and mouse embryonic fibroblasts [27, 28, 43]. In addition, endothelial-specific deletion of XBP1 decreased endothelial cell proliferation, reduces early stage retinal vasculogenesis and impairs angiogenesis under ischemic conditions [13]. These researches indicate that XBP1 plays a significant role in maintaining the physiological integrity of endothelial cells and pathological angiogenesis in ischemic tissues. Cardiac angiogenic imbalance leads to dilated cardiomyopathy, so spliced XBP1 may presents its cardioprotection against prolonged cardiac stress by promoting VEGF mediated-cardiac angiogenesis. XBP1 also regulates a variety of genes involved in cellular metabolism [44], redox state [45], autophagy [12], inflammation [46], cell survival [47]. Therefore, the nature of XBP1 in other models of cardiac hypertrophy and heart failure needs to be further investigated.

## Conclusions

Our study has for the first time established that XBP1 is an important angiogenic factor to maintain normal cardiac function in the early stage of hypertrophy and deregulation of of mir-214 and miR-30* in the hypertrophic and failing hearts inhibits XBP1 and XBP1-induced angiogenesis results in the transition of hypertrophic hearts into heart failure. Thus, mediation of miRNAs might be a valid therapeutic target to prevent heart failure.

## Additional file

10.1186/s12967-015-0725-4 Real-time PCR analysis of the ratio of miR-30a*/miR-214 in mice heart and VEGF and EDEM mRNA in siRNA-XBP1treated H9C2 cells and representative immunostaining of CD31 in hearts are presented.

## Abbreviations

ATF: activating transcription factor
ER: endoplasmic reticulum
Grp: glucose-regulated protein
IRE: inositol-requiring kinase
ISO: isoproterenol
AAC: abdominal aorta constriction
TAC: thoracic aorta constriction
UPR: unfolded protein response
VEGF-A: vascular endothelial growth factor-A
XBP: X-box binding protein
CHOP: C/EBP homologous protein
ANP: atrial natriuretic peptide
EDEM: ER degradation-enhancing α-mannosidase-like protein
