# Supplementary material for: MicroRNA regulation of unfolded protein response transcription factor XBP1 in the progression of cardiac hypertrophy and heart failure in vivo
Source: J Transl Med. 2015 Nov 16;13:363. doi: 10.1186/s12967-015-0725-4 (PMC4647486; doi:10.1186/s12967-015-0725-4)
Supplement: Supplementary file 1 — 10.1186/s12967-015-0725-4 Real-time PCR analysis of the ratio of miR-30a*/miR-214 in mice heart and VEGF and EDEM mRNA in siRNA-XBP1treated H9C2 cells and representative immunostaining of CD31 in hearts are presented. [file 12967_2015_725_MOESM1_ESM.ppt]

## Slide 1
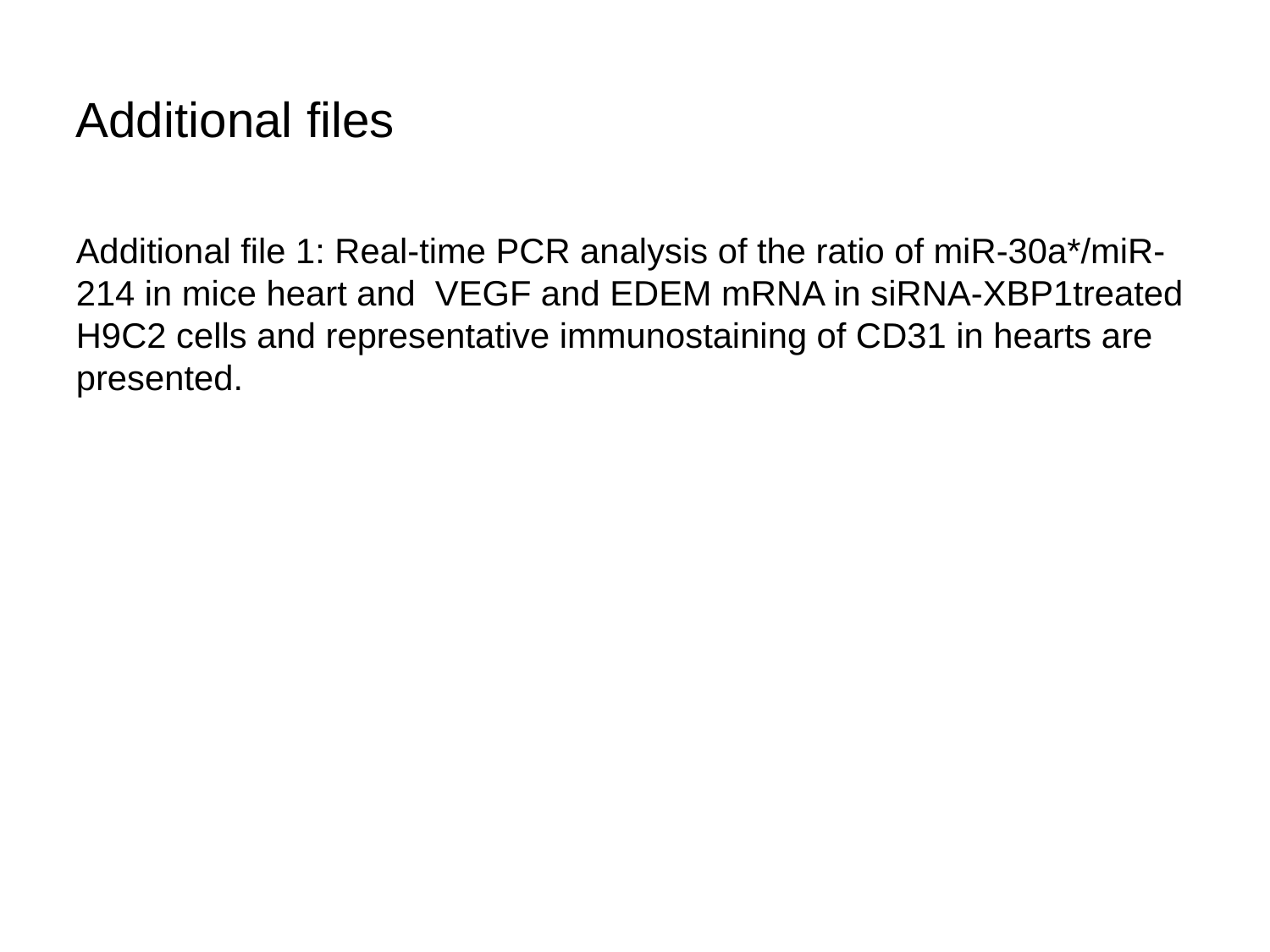

# Additional files
Additional file 1: Real-time PCR analysis of the ratio of miR-30a*/miR-214 in mice heart and VEGF and EDEM mRNA in siRNA-XBP1treated H9C2 cells and representative immunostaining of CD31 in hearts are presented.

## Slide 2
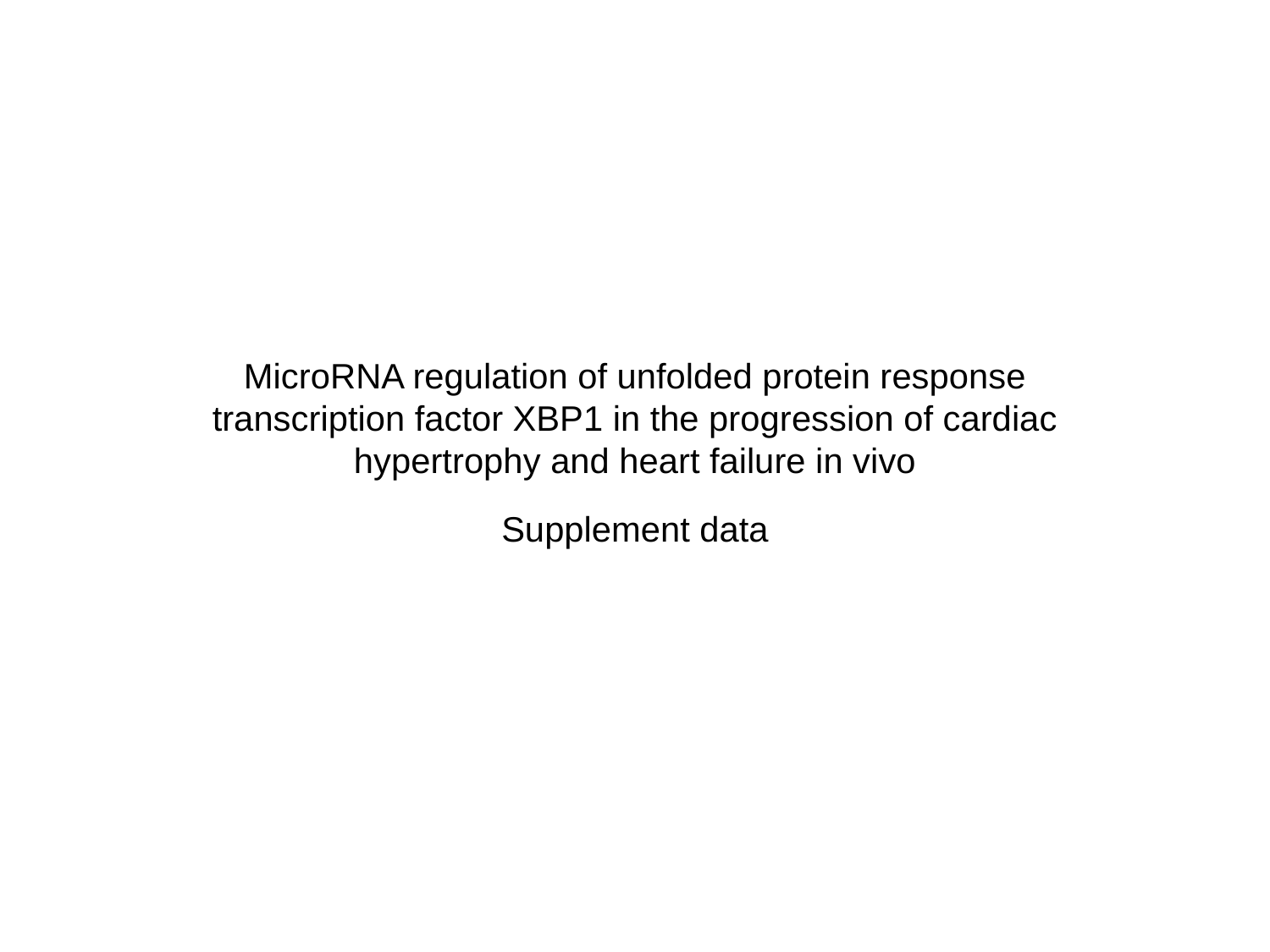

# MicroRNA regulation of unfolded protein response transcription factor XBP1 in the progression of cardiac hypertrophy and heart failure in vivo
Supplement data

## Slide 3
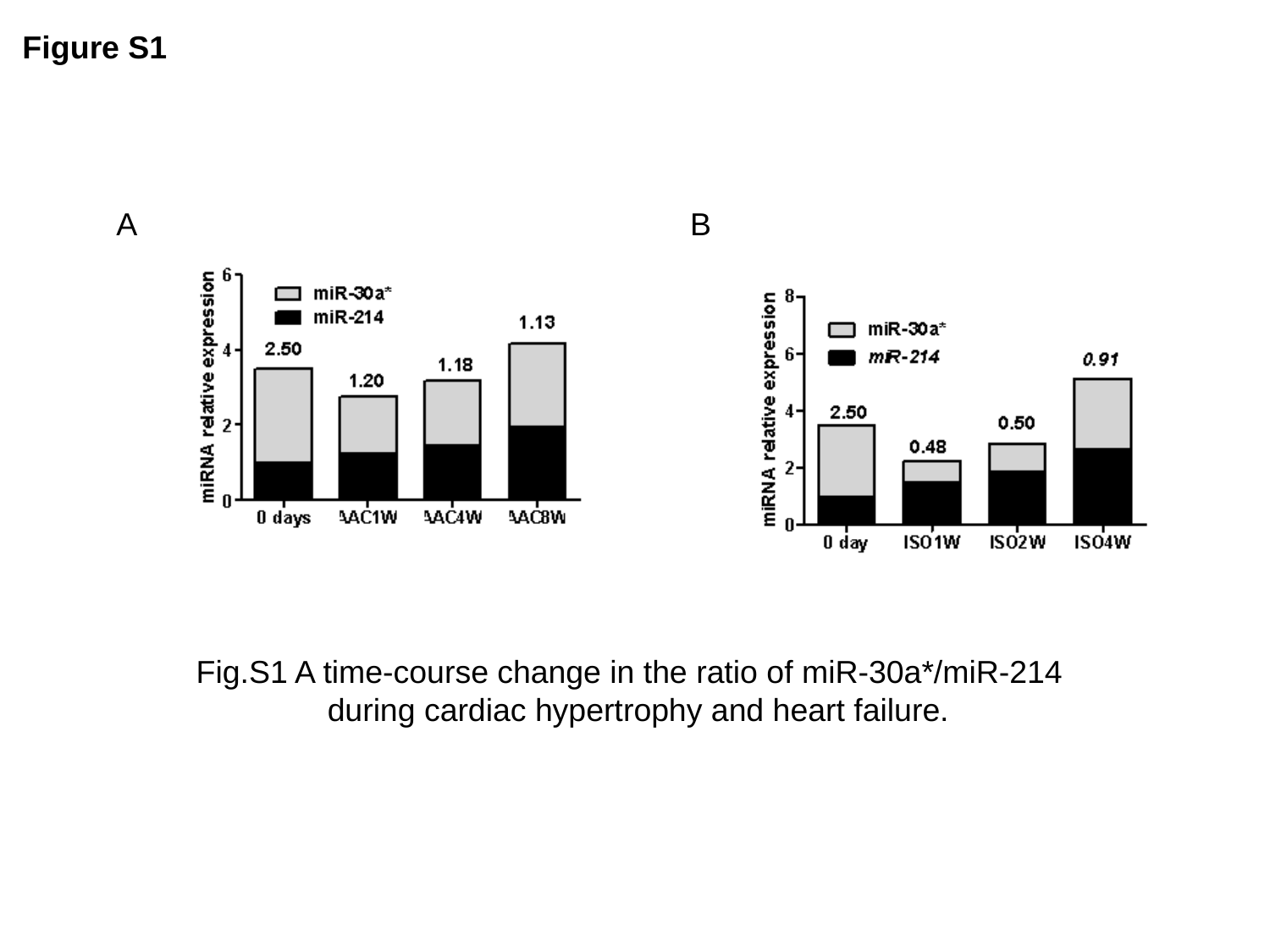

Figure S1
A
B
Fig.S1 A time-course change in the ratio of miR-30a*/miR-214 during cardiac hypertrophy and heart failure.

## Slide 4
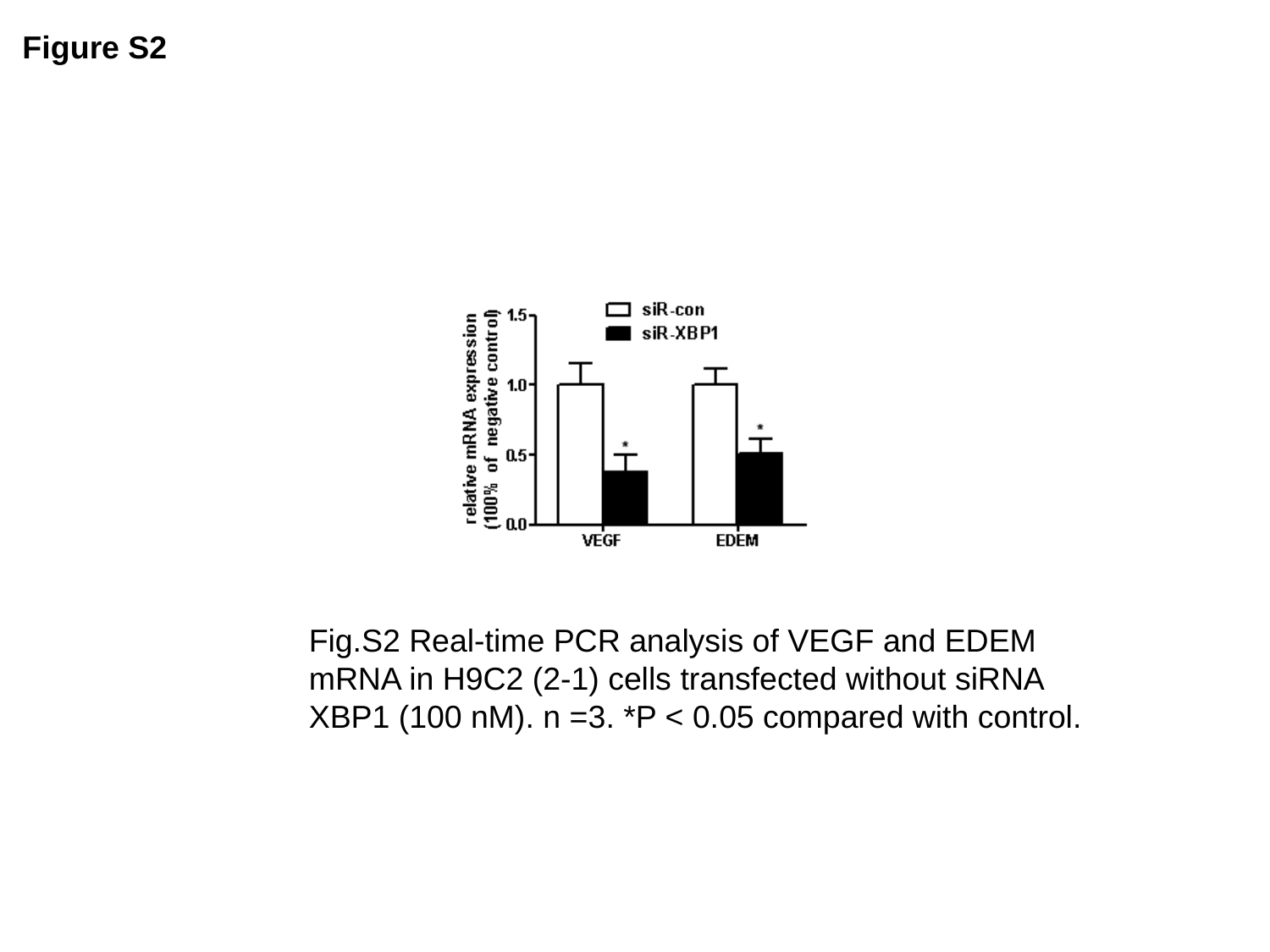

Figure S2
Fig.S2 Real-time PCR analysis of VEGF and EDEM mRNA in H9C2 (2-1) cells transfected without siRNA XBP1 (100 nM). n =3. *P < 0.05 compared with control.

## Slide 5
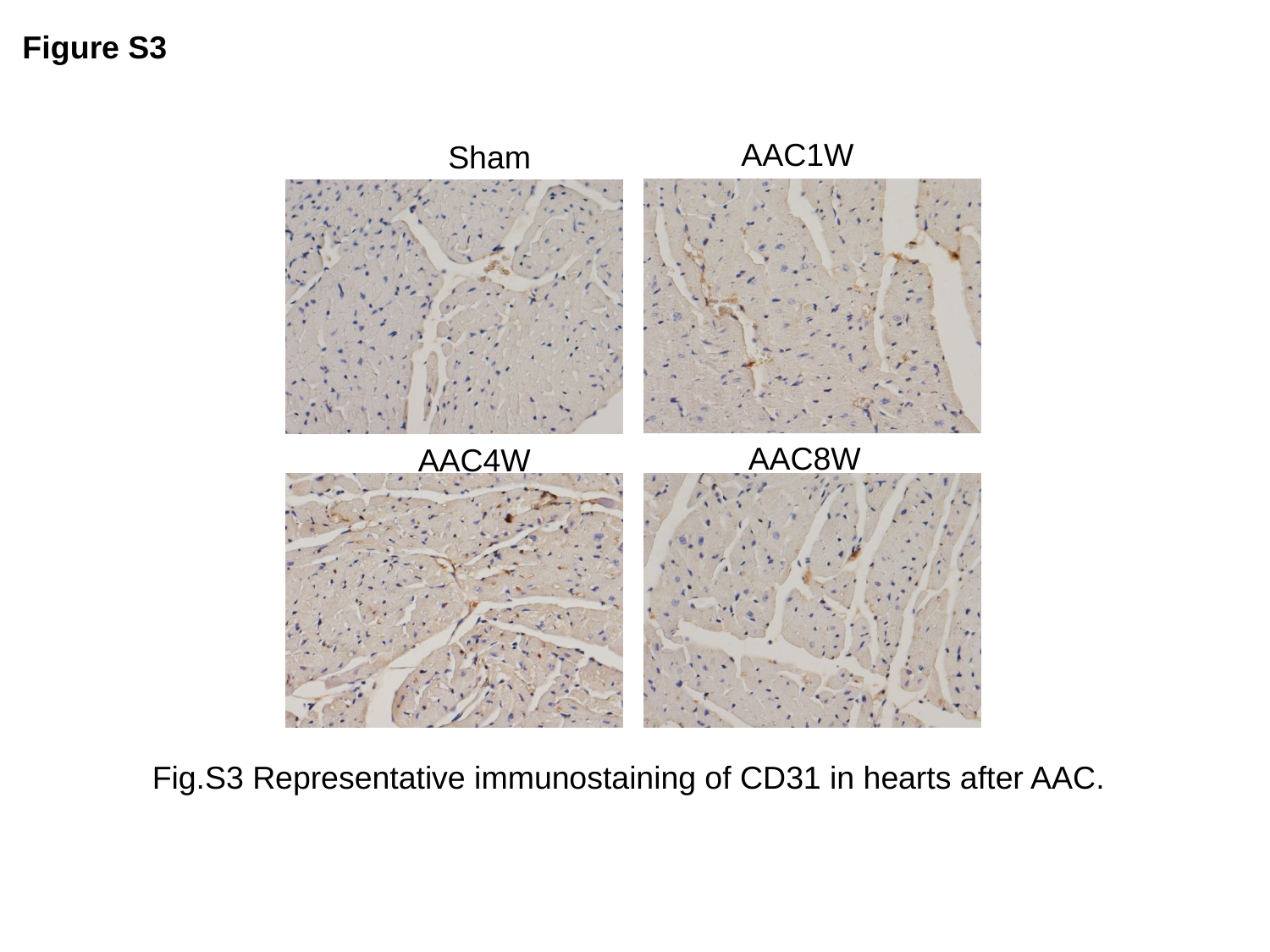

Figure S3
AAC1W
Sham
AAC8W
AAC4W
Fig.S3 Representative immunostaining of CD31 in hearts after AAC.
